# Novel Antennapedia and Ultrabithorax trimeric complexes with TBP and Exd regulate transcription

**DOI:** 10.1186/s41065-024-00327-x

**Published:** 2024-07-30

**Authors:** Alely Villarreal-Puente, Claudia Altamirano-Torres, Gustavo Jiménez-Mejía, Carolina Hernández-Bautista, Rubén Montalvo-Méndez, Martha Vázquez, Mario Zurita, Diana Reséndez-Pérez

**Affiliations:** 1 Facultad de Ciencias Biológicas, Departamento de Inmunología y Virología, Universidad Autónoma de Nuevo León, San Nicolás de los Garza, Nuevo León, México; 2 Facultad de Ciencias Biológicas, Departamento de Biología Celular y Genética, Universidad Autónoma de Nuevo León, San Nicolás de los Garza, Nuevo León, México; 3 Instituto de Biotecnología, Departamento de Fisiología Molecular y Genética del Desarrollo, Universidad Nacional Autónoma de México, Cuernavaca, Morelos México

**Keywords:** Antp, UBX, TBP, Exd, Trimeric complexes, BiFC-FRET

## Abstract

**Background:**

Hox proteins interact with DNA and many other proteins, co-factors, transcriptional factors, chromatin remodeling components, non-coding RNAs and even the extracellular matrix that assembles the Hox complexes. The number of interacting partners continues to grow with diverse components and more transcriptional factors than initially thought. Hox complexes present many activities, but their molecular mechanisms to modulate their target genes remain unsolved.

**Results:**

In this paper we showed the protein-protein interaction of Antp with Ubx through the homeodomain using BiFC in *Drosophila*. Analysis of Antp-deletional mutants showed that AntpHD helixes 1 and 2 are required for the interaction with Ubx. Also, we found a novel interaction of Ubx with TBP, in which the PolyQ domain of TBP is required for the interaction. Moreover, we also detected the formation of two new trimeric complexes of Antp with Ubx, TBP and Exd using BiFC-FRET; these proteins, however, do not form a trimeric interaction with BIP2 or TFIIEβ. The novel trimeric complexes reduced Antp transcriptional activity, indicating that they could confer specificity for repression.

**Conclusions:**

Our results increase the number of transcriptional factors in the Antp and Ubx interactomes that form two novel trimeric complexes with TBP and Exd. We also report a new Ubx interaction with TBP. These novel interactions provide important clues of the dynamics of Hox-interacting complexes involved in transcriptional regulation, contributing to better understand Hox function.

**Supplementary Information:**

The online version contains supplementary material available at 10.1186/s41065-024-00327-x.

## Introduction

Hox genes are master transcriptional factors that specify the antero-posterior axis of metazoans; they contain the highly conserved homeodomain (HD) responsible for DNA binding and target gene expression control [1, 2]. Despite the high structural and DNA-binding similarity of the HDs, homeoproteins achieve great levels of specificity to precisely regulate their target genes [3]. Such functional specificity is acquired via protein-protein interactions (PPIs), particularly, Extradenticle (Exd) interacts with homeoproteins through the YPWM motif, modifying their DNA-binding selectivity [4]. Several Hox interactors have been identified including diverse transcription factors (TFs), chromatin remodeling complexes, non-coding RNAs and even extracellular matrix [5]. Other important interacting partners are the homeoproteins themselves such as the homodimerization of Scr and AbdA as well as AbdA-Ubx heterodimerization in vivo [6, 7].

Transcription regulation by homeoproteins at the RNA Pol II basal machinery is of particular interest, and specific interactions have been identified. Antennapedia (Antp) interacts with BIP2 (also known as TAF_II_155, TAF3 in the *Drosophila* TFIID complex) through the YPMW motif, with TFIIEβ via the HD and with TBP through polyQ stretches [8–10]. Ubx is linked to the basal machinery by its direct interaction with the RNA Pol II through the N51 residue of the HD [11]. A systematic analysis revealed a Ubx tissue-specific interactome in *Drosophila melanogaster* embryos with partners including chromatin remodelers and translation regulators [12]. In addition, protein complexes have also involved homeoproteins, cofactors and General Transcription Factors (GTFs); Exd and Homothorax (Hth) interact with MEIS-PBX in vitro, and Antp forms trimeric complexes with TBP-TFIIEβ or TBP-Exd which also modulate the Antp transcriptional functions in living cells [10, 13]. All these data clearly point to diverse and tissue-specific Hox interactomes for functional specificity. The diversity of interactors as well as the complexity of Hox protein-protein associations plainly indicates that homeoproteins recruit specific GTFs or cofactors and arrange complexes to modulate their target genes during development.

Here, we dissected the interaction of Antp with Ubx showing that helixes 1 and 2 of Antp, as well as residue E19 of helix 1, are involved in the interaction. Also, a novel interaction of Ubx with TBP was established, in which the PolyQ domain of TBP is required for the interaction. Furthermore, we established the new Antp trimeric complexes with Ubx-TBP and Ubx-Exd, which reduced Antp transcriptional activity. By increasing the Antp and Ubx interactomes, our results provide important clues of Hox protein complexes dynamics involved in transcriptional regulation, and thus contribute to better understand Hox function in development.

## Results

### Antp and Ubx interaction through HD

**Fig. 1:**
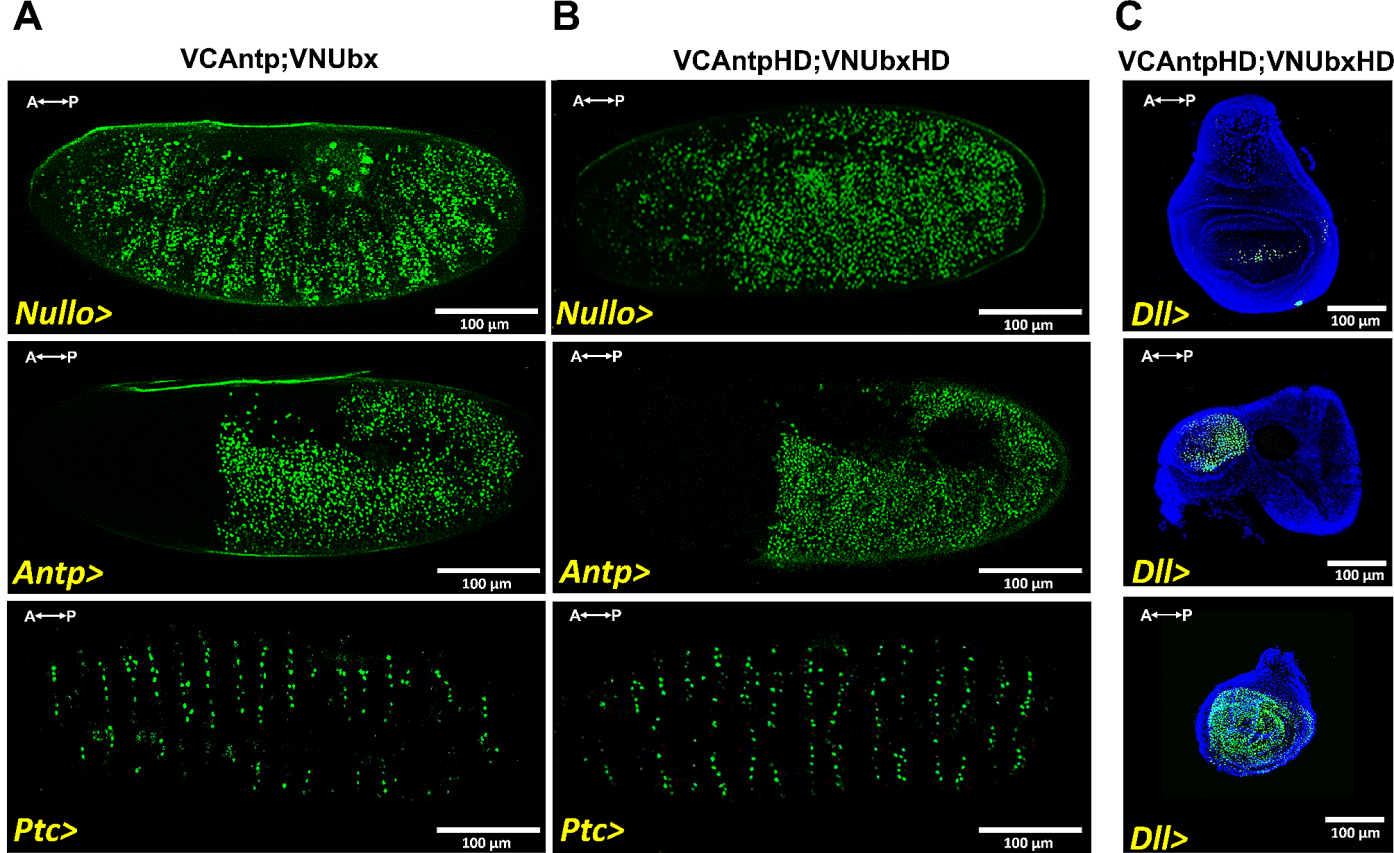
The Antp and Ubx HDs are directly involved in the interaction in *Drosophila*. **A**) Antp-Ubx FL interaction was detected by co-expression using *nullo-*, *antp-* and *ptc-*Gal4 drivers in embryos (green fluorescence). **B**) Interaction between Antp and Ubx HDs was detected in embryos. **C**) HDs Antp-Ubx interaction was detected in the eye-antenna, wing, and leg imaginal discs using the *dll*-Gal4 driver. DAPI staining (blue) was used for whole disc visualization. Anterior and posterior axes are indicated in the left superior corner. Scale bar, 100 μm

To determine the interaction between Antp and Ubx we performed Bimolecular Fluorescence Complementation (BiFC) in *Drosophila* embryos and imaginal discs using the UAS/Gal4 system. For BiFC assays, Antp and Ubx were fused either to N-terminal or C-terminal halves of the fluorescent protein Venus (VN and VC). The interaction of Antp with Ubx brings Venus fragments together, reconstituting the fluorescence. Co-expression of Antp and Ubx full-length (FL) proteins revealed interaction (Venus green fluorescence) in embryos under *nullo*-, *antp*– and *ptc*-Gal4 drivers (Fig. 1A). The HDs of Antp and Ubx showed BiFC fluorescence signal in embryos (*nullo*-, *antp*- and *ptc*–Gal4) and imaginal discs under *dll*–Gal4 indicating that the HDs are sufficient for the interaction in vivo (Fig. 1B-C). Our results clearly demonstrate that the HD is directly involved in Antp-Ubx interaction in *Drosophila*.

### Antp HD helix 1 and 2 are required for the interaction with Ubx

**Fig. 2:**
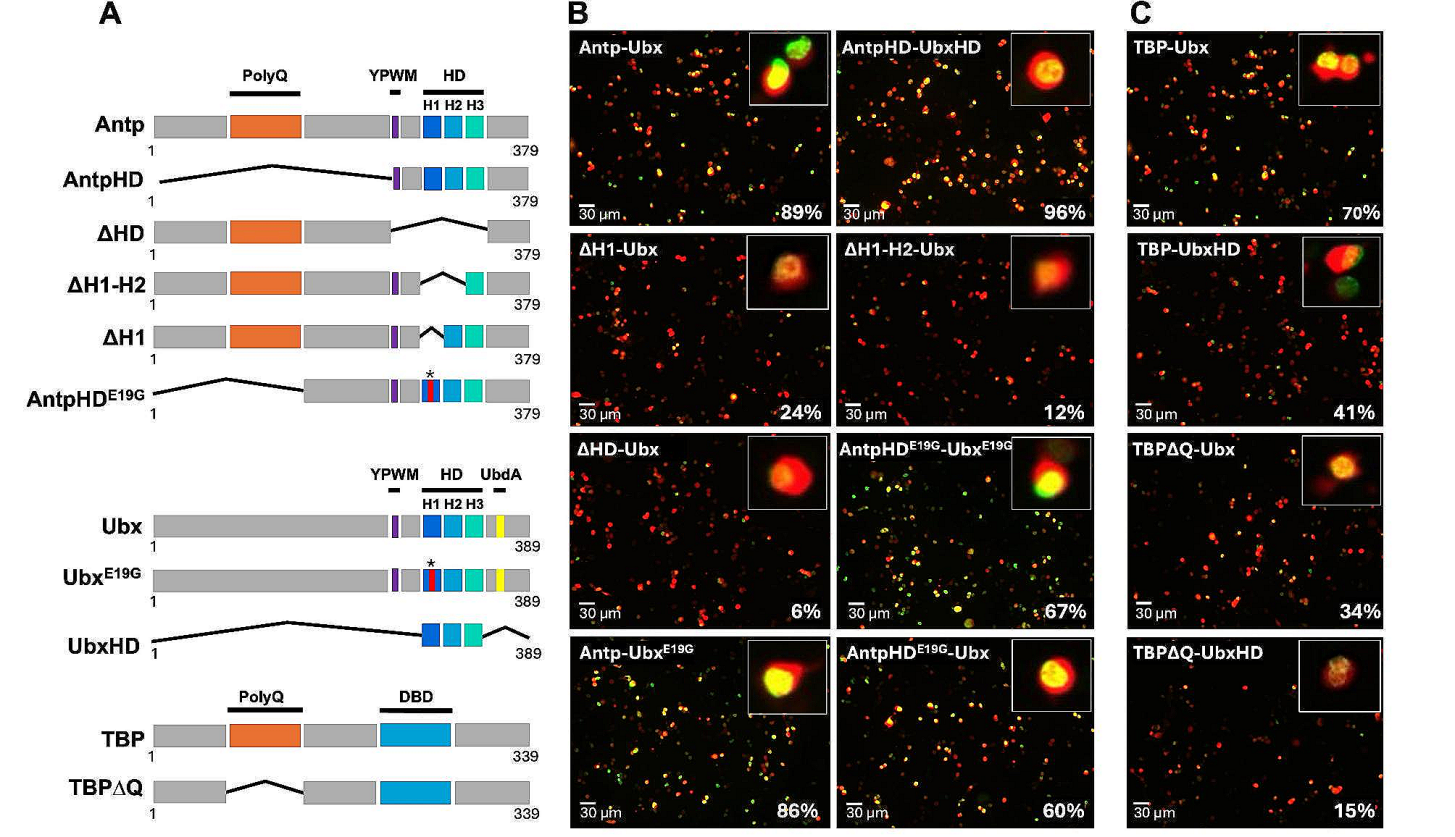
Antp HD helixes 1, 2 and PolyQ domain of TBP are necessary for interaction with Ubx. **A**) Schematic representation of the Antp, Ubx and TBP wild type and mutants cloned downstream of Venus halves in pCS2-VC155 (VC), and pCS2-VNm9 (VN). Antp, Ubx and TBP regions are indicated as PolyQ, YPWM motif, homeodomain (HD), UbdA motif and DNA binding domain (DBD). Deletions are represented as black lines and site-directed mutagenesis of helix 1 residue 19 with an asterisk (*). **B**) BiFC assays in the HEK293 cell line showing protein-protein interactions between Antp-Ubx and Ubx-TBP FL versions (Venus complementation in green). Antp deletion of HD or helixes 1 and 2 and Antp^E19G^ affect the interaction with Ubx, while Ubx^E19G^ version had no effect. **C**) Protein-protein interaction between TBP and Ubx by BiFC. Deletion of the N- and C-term of the Ubx as well as the PolyQ domain of TBP strongly affect the interaction. pCAG-mCherry was co-transfected as internal control (red fluorescence). Scale bar, 30 μm

Since the HD is required for Antp-Ubx interaction in vivo, we next performed BiFC assays in a HEK293 cell line to further analyze the putative domains involved in the interaction. To determine the regions required for the interaction, we used a battery of Antp and Ubx mutants previously reported (Fig. 2A and S2; 14,9) and the newly constructed Ubx^E19G^ (Fig. 2A). As expected, Antp-Ubx interaction was found in 89% of the transfected cells using FL proteins (Fig. 2B and S1A). Deletion of Antp HD (AntpΔHD) greatly decreased the interaction to 6% (Fig. 2B) compared to Antp-Ubx interaction (Fig. 2B and S1), indicating that the Antp HD is required for the interaction with Ubx in living cells. According to this, the HDs from both proteins (AntpHD and UbxHD) showed a significant increase of interaction to 96% (Fig. 2B and S1) compared to FL proteins. Next, we aimed to identify the specific region of Antp HD involved in its interaction with Ubx using Antp mutants. Absence either of helix 1 (AntpΔH1), or both helix 1 and 2 (AntpΔH1-2) significantly decreased the interaction with Ubx to 24% and 12% respectively (Fig. 2B and S1). These findings suggest that Antp HD helixes 1 and 2 are involved in this interaction.

Glutamic acid (E) at position 19 of the Antp HD is important for its functions and interactions. It was shown earlier that replacing the glutamate to glycine (G) interferes with the interaction between Hox and Pax proteins [7, 14]. The E is within the highly conserved HD motif TLELEKEF, which is shared by Antp and Ubx at identical positions. Therefore, we further analyzed the AntpHD^E19G^ and Ubx^E19G^ mutants in BiFC assays. The single mutant AntpHD^E19G^ showed a significant reduction of the interaction with Ubx to 60%, in contrast with Ubx^E19G^ which showed no effect in the interaction with Antp, maintaining 86% of BiFC positive cells (Fig. 2B and S1A), pointing out that Ubx E19 position is not required for this interaction. Co-transfection of both single mutants AntpHD^E19G^-Ubx^E19G^ showed an interaction of 67%, similar to AntpHD^E19G^, indicating that specifically the Antp position E19 on helix 1 is important for the interaction with Ubx.

We previously demonstrated Antp-TBP interaction by BiFC [10], and we wondered whether Ubx also interacts with TBP. BiFC assays in cell culture showed that TBP interacts with Ubx in 70% of transfected cells (Fig. 2C and S1B). Since the functional relevance of the N-terminal located PolyQ stretch in TBP has been previously demonstrated, as well as its role in TBP-Antp interaction, we also tested if the TBP version without the PolyQ stretch affected its interaction with Ubx; this TBP mutant showed a decrease in BiFC interaction to 34% (Fig. 2C and S1B). Additionally, as it has been previously reported that N-terminal regions in Ubx are essential for its transcriptional activity [15], we proved that UbxHD affected its interaction with TBP, decreasing to 41% (Fig. 2C and S1B). According to this, using both TBP∆Q and UbxHD mutant versions showed a significant decrease to 15% (Fig. 2C and S1B). Taken together, these results indicate that DNA binding domains in TBP and Ubx do not mediate the TBP-Ubx interaction, but rather their N-terminal domains as the PolyQ domain of TBP or the UbdA region in Ubx C-terminal.

### Antp forms trimeric complexes with Ubx and TBP

**Fig. 3:**
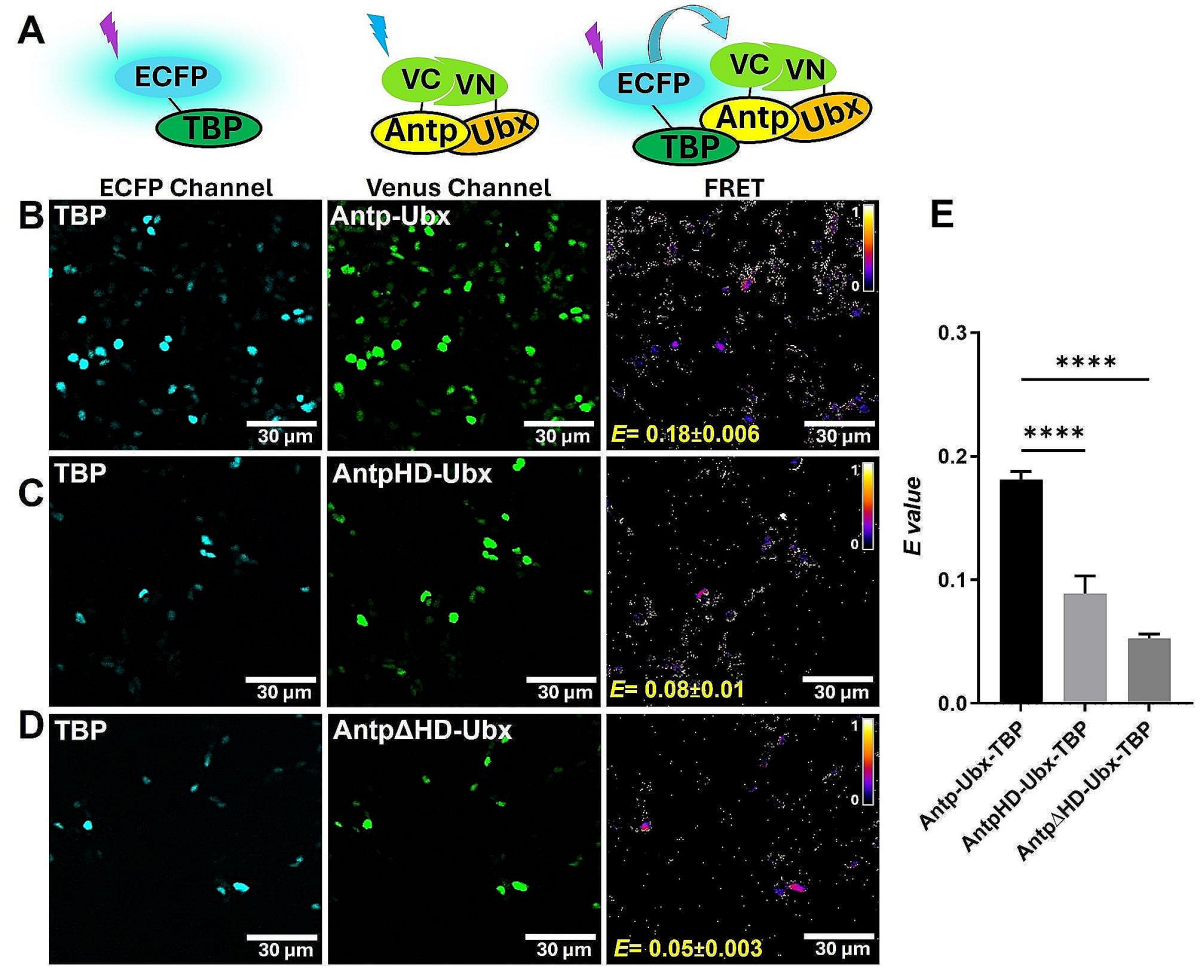
Trimeric interaction of Antp-Ubx with TBP using BiFC-FRET. **A**) Schematic representation of TBP fused to ECFP (ECFP Channel), BiFC by Antp-Ubx interaction (Venus channel) and energy transfer due to TBP-Antp-Ubx trimeric complex (FRET). **B**) TBP trimeric interaction with Antp-Ubx heterodimer (*E* = 0.18 ± 0.006). **C**) Absence of PolyQs in AntpHD diminished the trimer formation *(E* = 0.08 ± 0.01). **D**) AntpΔHD caused a reduction in the trimeric interaction (*E =* 0.05 ± 0.003). Color bar represents FRET signal intensity (Fire mode): brighter colors indicate high trimeric interaction levels and darker colors indicate low trimeric interaction level. Scale bar, 30 μm. **E**) Statistical analysis of three independent FRET experiments using a one-way ANOVA and Tukey for mean comparison, the high significance is indicated with **** (*p* < 0.0001), error bars correspond to standard error

Given that TBP interacts through its PolyQ region with Antp and Ubx, we next tested the hypothesis of a putative formation of trimeric complexes. We used the BiFC-FRET approach that was previously standardized in cell culture [10] with the reconstitution of the fluorescent protein Venus by BiFC interaction (VCAntp-VNUbx) used as acceptor and TBP fused to ECFP as donor (Fig. 3A). In a trimeric complex, the ECFP donor is in close proximity to the acceptor and catalyzes an energy transfer, which can be visualized by confocal microscopy as signal intensity and calculated and represented as an *E*-value. A trimeric complex of TBP with Antp-Ubx was clearly shown with a high *E* value of 0.18 ± 0.006 (Fig. 3B). To validate this trimer formation, we prevent the Antp dimeric interactions using specific mutants. Disruption of the Antp-TBP interaction by PolyQ absence in AntpHD caused a significant reduction of trimer formation (*E* = 0.08 ± 0.01; Fig. 3C and E). Similarly, when we disrupted the Antp-Ubx dimer with AntpΔHD there was significant reduction on the trimeric complex (*E* = 0.05 ± 0.003; Fig. 3D and E). The trimer *E* value declined due to interaction disruption that surely validated the Antp trimeric complex with Ubx and TBP in cell culture.

### Trimeric complexes of Antp with Ubx and Exd

**Fig. 4:**
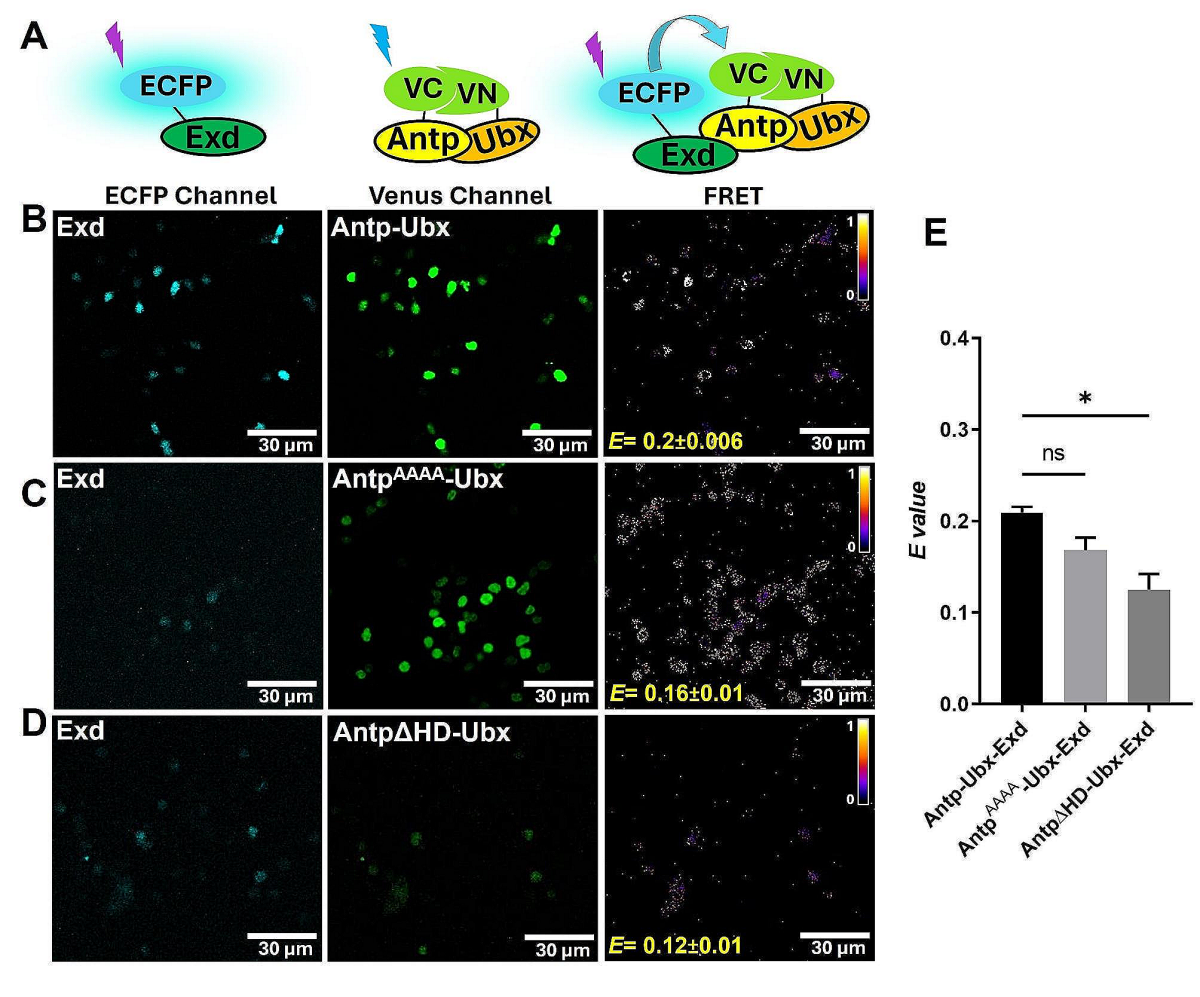
Exd forms trimeric complexes with Antp-Ubx by BiFC-FRET. **A**) Schematic representation of Exd fused to ECFP (ECFP Channel), BiFC by Antp-Ubx interaction (Venus channel) and energy transfer due to Exd-Antp-Ubx trimeric complex (FRET). **B**) Exd trimeric interaction with Antp-Ubx heterodimer (*E* = 0.2 ± 0.006). **C**) FRET due to Antp^AAAA^ (*E =* 0.16 ± 0.01) is shown (ns). **D**) AntpΔHD affected significatively the trimeric interaction (*E =* 0.12 ± 0.01). Color bar represents FRET intensity (Fire mode): brighter colors indicate high trimeric interaction levels meanwhile darker colors indicate low trimeric interaction levels, scale bar, 30 μm. **E**) Statistical analysis of three independent FRET experiments using a one-way ANOVA and Tukey for mean comparison, the significance is indicated with * (*p* < 0.05), Bars correspond to standard error

We also tested whether Antp-Ubx interacted in a trimeric way with Exd fused to ECFP as donor (Fig 4A). Results indicate the formation of an Antp-Ubx-Exd trimeric complex (*E* = 0.2 ± 0.006; Fig. 4B). To confirm this interaction, we disrupted the Antp-Exd dimer using the YPWM mutant Antp^AAAA^; the FRET reduction is not significant compared to Antp-Ubx-Exd complex (*E* = 0.16 ± 0.01; Fig. 4C and E), however, this result is coherent with the fact that Exd also interacts with Ubx [16]. Conversely, the AntpΔHD mutant that impedes Antp-Ubx dimer also showed a significant decrease of FRET signal (*E* = 0.12 ± 0.01; Fig. 4D and E). Our results confirmed that Exd forms trimeric complexes with Antp and Ubx.

In contrast, we did not detect formation of Antp-Ubx trimeric complexes with BIP2 and TFIIEβ. The BiFC-FRET analysis showed BIP2 and TFIIEβ *E* values of 0.08 ± 0.005 (Fig S3 A-B) and 0.09 ± 0.001 respectively (Fig S3 C-D), indicating absence of trimeric complexes due to the lack of energy transfer.

### Trimeric complexes reduced transcriptional activity of Antp

**Fig. 5:**
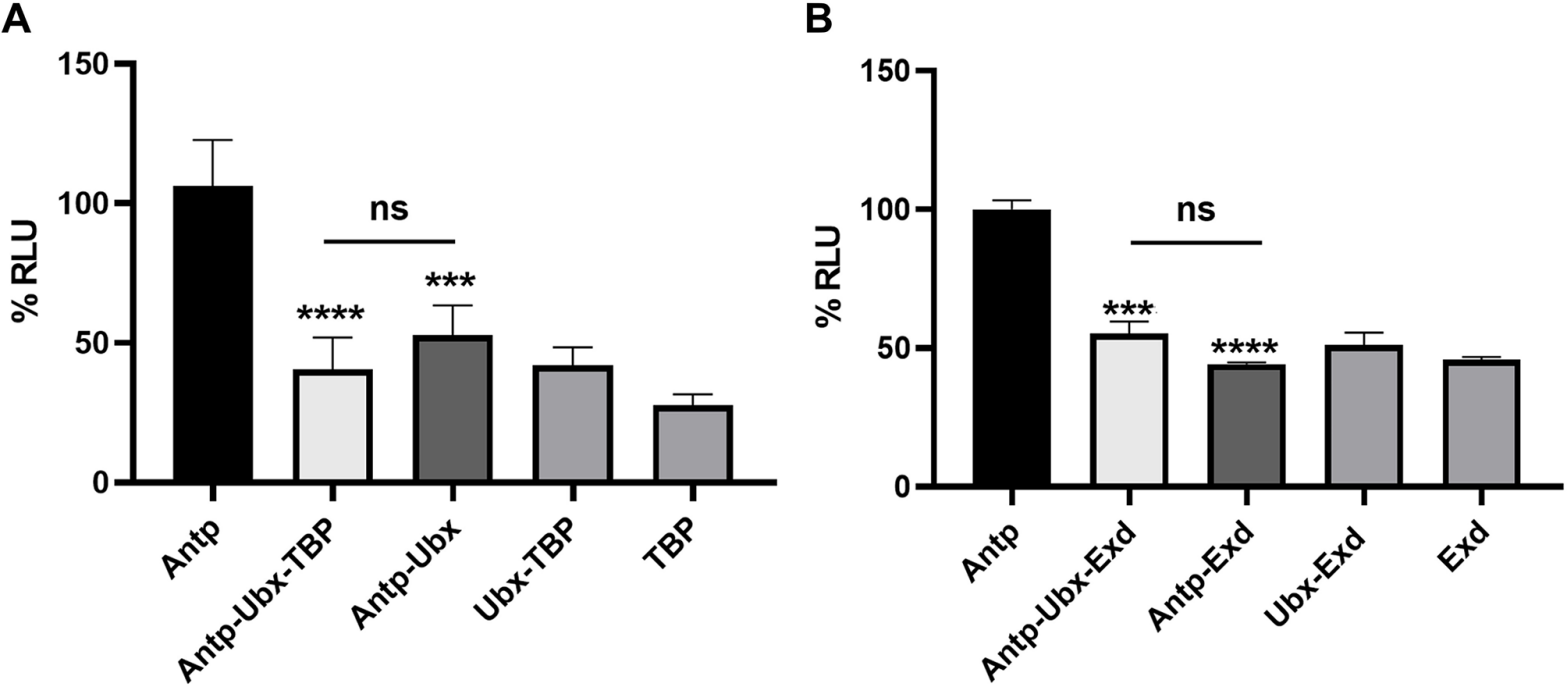
Trimeric complexes of Antp-Ubx with TBP or Exd affect Antp transcriptional activity. The graphic shows the percentage of Antp transcriptional activity to binding BS2 sites (% RLU). **A**) Antp-Ubx-TBP trimer as well as the dimers diminish transcriptional function of Antp. **B**) Antp-Ubx-Exd and the dimers Antp-Exd and Ubx-Exd decrease Antp transcriptional activity. Statistical analysis was made by one-way ANOVA with the post-hoc Tukey for mean comparison in three independent experiments. Error bars correspond to standard error (*p* < 0.0001)

To determine the effect of the complexes on Antp transcriptional activity, we used a luciferase (LUC) reporter (pGLH11) with a minimal Hsp70 promoter and eleven oligomerized BS2 Antp-binding sites previously described in HEK293 cells [17]. The Antp-Ubx-TBP trimer showed a statistically significant reduction of transactivation to 30.93 ± 10.52% compared to Antp. In a similar fashion, the Antp-Ubx dimer also had a significant reduction of LUC activity to 43.88 ± 11.60% (Fig. 5A). There is no significant difference between the transcriptional activity of the Antp-Ubx-TBP and Antp-Ubx complexes, indicating that both the trimeric and dimeric complexes affected the Antp transactivation activity. We also found that Ubx has a transactivation activity of 53.47 ± 10.36%, significantly different than Antp; however, when Ubx activity is compared to the Antp dimer and trimer activities there is no significant difference (data not shown). These results indicate that the Antp transactivation function is specifically affected by these complexes. Similarly, the Antp-Ubx-Exd trimer decreases the transactivation activity of Antp to 57.94 ± 4.74%, which is statistically significant compared to Antp. We also found a significant reduction of the Antp transcriptional activity in the Antp-Exd dimer to 45.87 ± 2.42% (Fig. 5B). Overall, the transactivation assays indicate that Antp trimeric and dimeric interactions with Ubx, TBP or Exd have a down-regulating effect in the Antp transcriptional activity.

## Discussion

Here, we showed that the dimeric interaction of Antp with Ubx is through Antp HD helixes 1 and 2 as well as residue E19 of helix 1. We also report a novel interaction of Ubx with TBP in which the PolyQ domain of TBP is required in this interaction. Furthermore, we established the two new Antp trimeric complexes with Ubx-TBP and Ubx-Exd and both trimers are important for Antp transactivation activity in living cells.

Our results demonstrate that the HDs of both Ubx and Antp are sufficient for maintaining the interaction in embryos. It has been proven that the HD is enough for other protein-protein interactions, for example, the interaction of Antp with the transcription factor Eyeless (Ey) in *Drosophila* also depends on their HDs [14]. Another report indicates that the Scr homodimerization depends on the HD and is required for Scr function in *Drosophila* [7]. The HD is also required for the Antp interaction with the basal transcription machinery factor TFIIEβ in cell culture and in vivo [9].

The HDs showed stronger interaction signal compared to the FL proteins, suggesting that the regions upstream or downstream of the HD may interfere dimerization in FL proteins as previously observed in Scr, Antp, Ubx, AbdA and AbdB interactions with other TFs in living embryos [18]. A similar effect was observed with the HD of AbdA which showed a strong interaction with Med19 in *Drosophila* embryos, whereas the FL AbdA is not able to interact with Med19 [19]. In both reports, a similar expression level of HD or FL proteins was confirmed, suggesting that the observed interaction dynamics are not due to overexpression. In addition, in vivo functional analysis of Antp HD peptides showed almost equal levels of the endogenous Antp protein [17]. Hence, the stronger interaction levels showed by the HDs are most likely due to absence of HD-adjacent regions. This evidence clearly indicates the HD’s sufficiency as a platform for protein-protein interactions, as well as the inhibitory activity of different structural motifs along the Hox proteins.

Both HD helixes 1 and 2 are involved in the Antp-Ubx interaction. Other reports indicate the HD helix 1 as responsible for the homeoprotein interactions between Antp-Ey and Scr-Scr, and that the E19 helix residue is key for these interactions [7, 14]. In addition, the HD helix 1 of HOXD8 mediates its interaction with HOX9 as well as its transcription activities [20]. Accordingly, our results also pointed out the specific relevance of the Antp E19 residue for its interaction with Ubx. Other Ubx HD residues cannot be ruled out, since it was previously found that Ubx HD residues 23 and 25 on helix 1, and 57 on helix 3 are important for the in vitro interaction with the cofactor Exd, and the residues 22 and 24 are highly conserved and positionally exposed in the Ubx HD [21, 22].

Antp residues 32 and 36 of HD helix 2 were not required in the Antp-Ubx interaction (data not shown), although they are important for interaction with the transcription factor TFIIEβ [9]. However other aminoacidic residues in Antp helix 2 could be involved because it was previously reported that HD residues 30 and 33 of POU factors Oct-1 and Oct-2 are relevant for their interaction with the Herpes simplex virus transactivating factor VP16. Also, residues 29 and 56 of the HOXA9 HD have a redundant function with the HX motif for interaction with PBX1 and MEIS1 [23–25]. Overall, these results support the importance of Antp position E19 on helix 1 for the interaction with Ubx.

Previous reports have indicated the interaction of homeoproteins like CDX1, Sp1 and Antp with the basal transcription factor TBP [10, 26, 27]. Accordingly, we confirmed for the first time that the interaction of Ubx with TBP was found to be dependent on the PolyQ regions of TBP and the N- and C-term of Ubx including the UbdA domain, which could play a role in the interaction. The PolyQ region of TBP has been extensively corroborated as an interaction platform for other factors [10, 28, 29]. The Ubx N-term regions do not contain PolyQ stretches, however, it has been previously reported that the Ubx N-term is essential for its transcriptional activity [15], and the intrinsically disordered regions have proven to be necessary for Ubx protein interactions [12, 30]. The link of homeoproteins interacting with the RNA Pol II basal machinery has been established numerous times with other GTFs like TFIIEβ [9, 20, 31] and the transcription pausing factor M1BP [32].

We found that Ubx diminished the Antp transactivation activity; similarly, other HD factors such as AbdB, Ey and Scr also reduced Antp transcriptional activity [14, 33]. It is well known that Ubx represses the expression of Antp in the 3rd thoracic segment of the embryo [34, 35]. Our transactivation findings with Antp and Ubx likely suggest that, in addition to promoter binding, protein-protein interactions may be a part of the transcriptional regulation mechanisms of homeoproteins for posterior prevalence in the embryo. The transcriptional activity of Antp was not rescued by absence of HD helixes 1 and 2 (data not shown), similar to the report of Cárdenas-Chávez in 2012 where the Antp helix 1 mutant E19G decreased the interaction with AbdB but did not rescue the transcriptional activity.

Although the transactivation system with 11 oligomerized BS2 sites is specific for Antp, some level of Ubx transactivation was observed with this system (data not shown), however this activity differs from Antp, indicating the BS2 sites are Antp-specific. Other homeoproteins such as Exd, HoxB7, HoxB8 and HoxC8 also recognize the BS2 binding sites, but it was not determined whether there is transcriptional activity [36].

We also present the first report of trimeric protein complexes of Antp-Ubx with TBP or Exd using the previously standardized BiFC-FRET assay [10]. The trimer reduction using the Antp PolyQ or HD mutants corroborates the formation of the Antp-Ubx-TBP trimer and supports the key role of Antp PolyQ regions in the interaction with TBP previously described [10], as well as the importance of the Antp HD for its interaction with Ubx. We also validated the trimer complex of Antp-Ubx with Exd using mutants that specifically disrupted the Antp-Ubx or Antp-Exd dimers. Mutation of the Antp YPWM motif did not significantly reduce the *E* value, since the Ubx YPWM motif was not mutated; these results are in accordance with the fact that Antp and Ubx both interact with Exd through this motif [3, 7, 37, 38]. In this scenario, Ubx probably keeps Exd close enough for the energy transfer to occur; this is also feasible due to evidence indicating that Ubx has multiple interactions with Exd besides the YPWM motif, such as the UbdA motif [39, 40]. This also indicates that Antp-Ubx-Exd trimer is formed by multiple interacting domains involving the HDs and the YPWM motif. This has been reported in the trimeric interactions of Antp-Exd or Ubx-Exd with Hth [41] and, in the Antp-Ubx-Exd trimer, raising the possibility of wider complexes involving other transcriptional factors for gene regulation.

The absence of trimeric interactions of Antp-Ubx either with BIP2 or TFIIEβ corroborated with Antp mutants (data not shown) is similar to the absence of Antp-TBP trimeric interaction with BIP2 previously described using BiFC-FRET [10]. This adds to the dynamism of Hox proteins interactions, since BIP2 seems to be involved mainly in dimeric interactions whereas other GTFs like TFIIEβ are required for some complexes like Antp-TBP and dispensable for others such as Antp-Ubx.

The trimeric complexes of Antp-Ubx with TBP or Exd both showed a significant decrease of the Antp transcription activity in cell culture; the reduction is lower and maintained regardless of a partial Ubx and Exd LUC activity. A similar repression function has been determined for the trimers MES1-PBX-HOXA9 in myeloid leukemia and the Ubx-Exd-Hth in vivo [41, 42]. When we evaluated the function of Ubx using BS2 sites, we found that it is not affected by the formation of dimeric or trimeric complexes (data not shown). Overall, our results demonstrate two novel Antp trimeric complexes with Ubx-TBP and Ubx-Exd, which also regulate Antp gene transcription activity, indicating repression function in the trimer assembling.

Our results clearly showed that homeoproteins interact with each other as well as with multiple cofactors and GTFs in dimeric and trimeric ways, raising the question of how these complexes function at the transcriptional level during development. The Antp novel trimers here depicted diminished Antp transcription, indicating repression functions for these ensembles. In the Antp and Ubx interaction with TBP, the Hox proteins could prevent TBP and TFIIB interaction, since TBP acts as the TFIIB recruiter to the RNA Pol II PIC [43], therefore the trimer could be preventing the PIC assembly and therefore transcription initiation.

The Antp-Ubx interaction with the cofactor Exd adds a new interacting dynamic to the extensive evidence of complexes between Hox and the TALE (three amino acid loop extension) family of cofactors. Antp promotes leg identities in the thorax by repressing the activity of antennal- and head-determining genes such as Spalt, Hth and Dll and activating leg-specifying genes [44, 45]. Ubx specifies haltere identities by repressing the wing genes Dpp and Wg in the third thoracic segment [46, 47] and represses Antp promoter, preventing Antp expression towards the third thoracic segment.

Antp-Ubx interaction may function as an alternative in which both homeoproteins sequester themselves, preventing their respective binding to DNA in the limits of the 2nd and 3rd thoracic segments when both are present. Plainly, Antp and Ubx both have activation and repression activities, they can bind identical DNA motifs and even regulate common targets [48–50]. It is reasonable to think that the Antp-Ubx trimeric complex with Exd could be involved in the regulation of Antp and Ubx common targets, conferring specificity for repression.

Clearly, homeoproteins have versatility not only of functions but also of interactions, since Ubx also interacts with nuclear export factors like Embargoed (Emb) for autophagy repression in *Drosophila* [51]. The protein complex dynamics presented could also be extrapolated to the rest of Hox cellular activities, apart from gene regulation.

Hox proteins association with the RNA Pol II PIC are well known since the first report by Plaza et al., [14] and they continue to be described to date, either within dimer or trimer complexes [10, 52]. Apart from transcriptional activity, Hox ensembles with TBP, TFIIEβ or other GTFs [10] may also be participating in other RNA Pol II functions like transcription pausing during early development. Ubx and AbdA collaborate with the pausing factor M1BP, changing chromatin status to enable transcription [32]. Hunt et al., established that pausing release is triggered in a tissue-specific manner by enhancer regulation of Pol II primed promoters [53]. Thus, Antp-Ubx trimeric complexes may participate in linking the enhancer to the primed promoter via GTFs or pausing factors, orchestrating rapid transcriptional bursts.

Recently, Bandau et al., described a new RNA Pol II role for the reorganization of chromatin right after DNA replication, stabilizing several proteins including chromatin remodelers, histone modifiers and transcription factors [54]. Exd and Hth collaborate with Hox proteins for chromatin accessibility [55]; Ubx is involved in both opening and closing chromatin functions in vivo [56]. Therefore Antp-Ubx trimers either with TBP or Exd could collaborate with the RNA Pol II at chromatin level participating in remodeling tasks.

## Conclusions

In conclusion, we presented two novel trimeric complexes of Antp-Ubx with TBP and Exd that regulate Antp transcriptional function, opening the possibility of the regulation of Antp and Ubx targets and conferring specificity for repression. Our results increase the transcriptional factors in the Antp and Ubx interactomes and this variety of functions and interaction dynamics could be further analyzed during embryo development and extrapolated to other Hox proteins in Drosophila and even in mammals.

## Methods

### BiFC assays in Drosophila embryos

Fly crosses for BiFC assays were incubated at 25 °C overnight and the embryos were incubated at 4 °C for 48 h prior to BiFC visualization, according to Hudry et al., 2011 [6] Embryos were dechorionized with 1.5% sodium hypochlorite, washed with PBX buffer and mounted on slides with 60% Glycerol/PBS. Imaginal discs were dissected on PBS and mounted on slides using Vectashield mounting medium with DAPI (Vector laboratories, Southfield, MI, USA). Fly stocks were maintained at 18–25 °C on standard yeast-agar-cornmeal medium. Transgenic lines were kindly donated by Samir Merabet (UAS-VCAntp, VNUbx, -VNUbxHD and *antp*-Gal4) The drivers *ptc*- and *nullo*-Gal4 were purchased from Bloomington Stock Center.

### Plasmid constructs

For BiFC assays, Ubx and UbxHD coding sequences were amplified by PCR from genomic DNA of UAS-Ubx transgenic flies (Bloomington Stock Center). The ORFs were cloned in the pCS2VNm9 vector in frame with the coding sequence of the N-terminus of Venus (VN) using *Age*I and *Xba*I restriction sites. AntpHD^EG19^ and Ubx^E19G^ mutants were obtained by site-directed mutagenesis (Quickchange II XL kit, Stratagene, La Jolla, CA, USA). Antp, AntpHD, AntpΔHD, AntpΔH1 and AntpΔH1-2 in pCS2VC155; TBP and TBPΔQ in pCS2VNm9 were previously obtained [9, 10]. For BiFC/FRET assays, TBP coding sequence was amplified by PCR and cloned in frame with ECFP into pECFP-N1 using *Apa*I and *Age*I sites enzymes. pECFP-N1-Exd, -BIP2 and -TFIIEβ vectors were previously obtained [10]. Oligonucleotides sequences are available upon request. All plasmid constructions were verified by DNA sequencing before cell co-transfections.

### BiFC and transactivation assays in cell culture

HEK293 cells were cultured in DMEM (Dulbecco’s Modified Eagle Medium) supplemented with 10% FBS (Invitrogen, Carlsbad, CA. USA) and 1% penicillin-streptomycin (Sigma-Aldrich, Saint Louis, MI, USA). 1 × 10^5^ cells were seeded on 6-well plates with glass coverslips, cultured for 24 h co-transfected with 6 µg of plasmidic DNA using polyethylenimine (PEI) 15 mM (Sigma-Aldrich, Saint Louis, MI, USA), according to the manufacturer’s instructions. All BiFC co-transfections included the VN- and VC- plasmids along with pCAG-mCherry (donated by Ataúlfo Martínez-Torres) for BiFC and calculation of transfection efficiency percentage. The fluorescence signals were visualized 48 h after transfection using the Zeiss Axio Imager 2 microscope (Carl Zeiss, Germany). BiFC Interaction percentages were calculated by counting the number of Venus fluorescent cells in one hundred red fluorescent cells, with three independent triplicates.

For transactivation assays, HEK293 cells were seeded in 24-well plates and co-transfected as described above using different combinations pPAC plasmids, pGLH11 reporter and pcopia-βGal (to normalize the luciferase activity) as previously described [9, 10]. Luciferase values were obtained 48 h after transfection using the Dual-Luciferase Reporter Assay System Kit (Promega, Madison, WI, USA) according to the manufacturer’s instructions. The transfections assay was performed in three independent experiments by triplicate.

### BiFC-FRET assays

The HEK293 cells for BiFC-FRET assays were maintained under standard culture conditions. For transfection, cells were seeded on 6-well plates and transfected 48 h after using PEI 15 mM (Sigma-Aldrich, Saint Louis, MI, USA), according to the manufacturer’s instructions. To correct for bleed-through, Venus (BiFC) and ECFP plasmids fusion constructs were individually transfected. The cyan (donor) and venus (acceptor) fluorescent signals were visualized 48 h after transfection using an immersion objective on an Olympus BX61W1 confocal microscope and the Fluoview 4.0 software (Olympus, Tokyo, Japan). Ten-nanometer size photographs were collected in spectral mode (420–660 nm) using 10 nm of step size under the confocal parameters 600v, 1X gain and 10% laser potency with 20X objective. All images captured were analyzed in ImageJ software using FRETTY plug in; BiFC-FRET quantification (*E*-value) were carried out according to Jimenez-Mejia et al., 2022 [10].

### Electronic supplementary material

Below is the link to the electronic supplementary material.

Supplementary Material 1

## Data Availability

Not applicable.

## Abbreviations

BiFC: Bimolecular Fluorescent Complementation
FRET: Fluorophore Resonance Energy Transfer
ECFP: Enhanced Cyan Fluorescent Protein
Antp: Antennapedia
Ubx: Ultrabithorax
TBP: TATA-binding protein
VN: N-terminal region of Venus fluorescent protein
VC: C-terminal region of Venus fluorescent protein
