# Supplementary material for: Novel Antennapedia and Ultrabithorax trimeric complexes with TBP and Exd regulate transcription
Source: Hereditas. 2024 Jul 30;161:25. doi: 10.1186/s41065-024-00327-x (PMC11290222; doi:10.1186/s41065-024-00327-x)
Supplement: Supplementary file 1 — Supplementary Material 1 [file 41065_2024_327_MOESM1_ESM.docx]

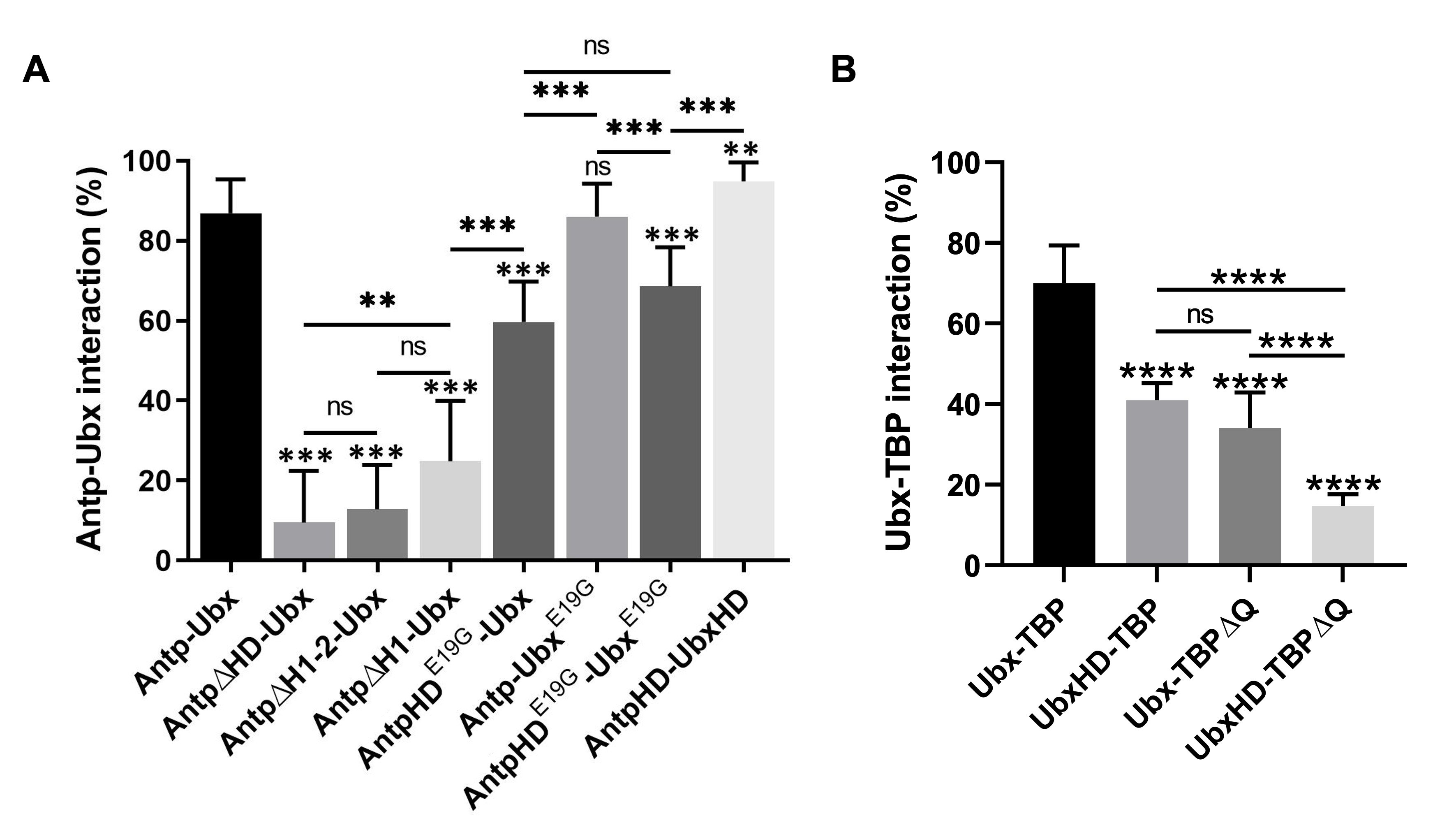
**Figure S1. Antp-Ubx and Ubx-TBP interaction in HEK293 cells by BiFC**. The percentage of interaction was determined by quantifying the Venus fluorescent cells in relation to the number of cells with the red fluorescent reporter mCherry. A) Statistical analysis of BiFC between Antp-Ubx interaction and their mutant versions. Mutations in Antp (AntpΔHD, AntpΔH1-2, AntpΔH1, AntpHD^E19G^) affect its interaction with Ubx significantly (***), whereas Ubx^E19G^ mutants show no significant difference (ns). B) Statistical analysis of BiFC interaction between Ubx and TBP and their mutated versions. The absence of Ubx N- and C-term as well as the PolyQ region of TBP decreases significatively the BiFC percentage (****) independently and in the same extent (ns). Also, when both mutated versions are tested the BiFC percentage decreases even more. Quantification of BiFC percentage between Antp-Ubx and Ubx-TBP were performed from three independent experiments. Statistical analysis was performed using a one-way ANOVA and the post hoc test Tukey for mean comparison (p < 0.05).



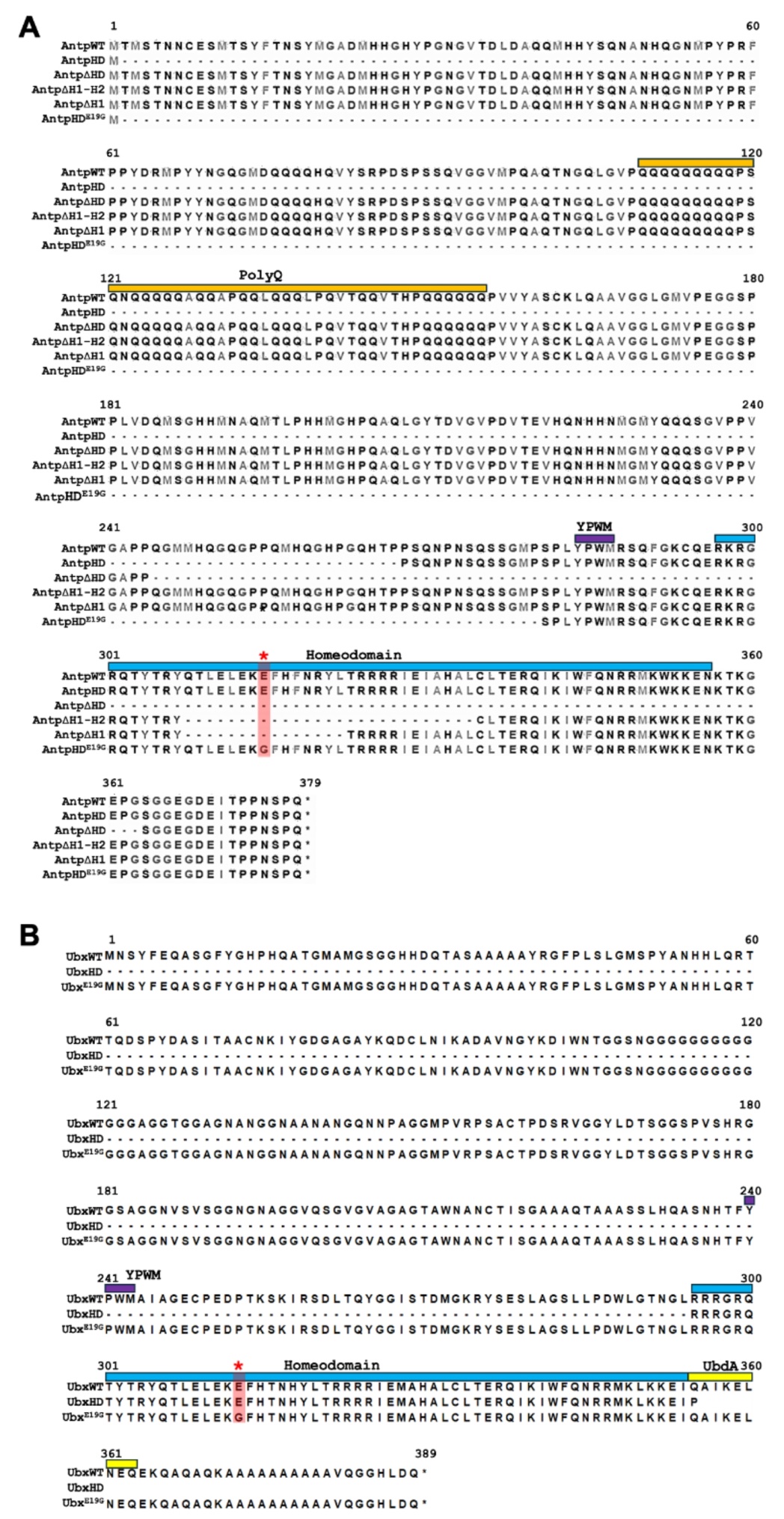



**Figure S2. Alignment of Antp and Ubx mutants. A)** Comparison of Antp WT amino acids sequences with Antp mutants: AntpHD deletion of amino-terminal (1-269), AntpΔHD lacking HD (245–363), AntpΔH1-H2 with deletion of 1 and 2 helixes (308–334), AntpΔH1 without H1 helix (308–322), and AntpHD^E19G^ lacking amino-terminal region and substitution of E to G at 19 position of HD region. B) Ubx WT amino acids sequence compared to UbxHD (295–355) corresponding to N- and C-term deletion, and Ubx^E19G^ with a change of position 19 of E to G in HD helix 1. Antp PolyQ region is indicated in orange, YPWM motif in purple, homeodomain in blue, and Ubx UbdA motif in yellow. Dashes indicate deletions, and the substitutions E19G are indicate in red with an asterisk (*).


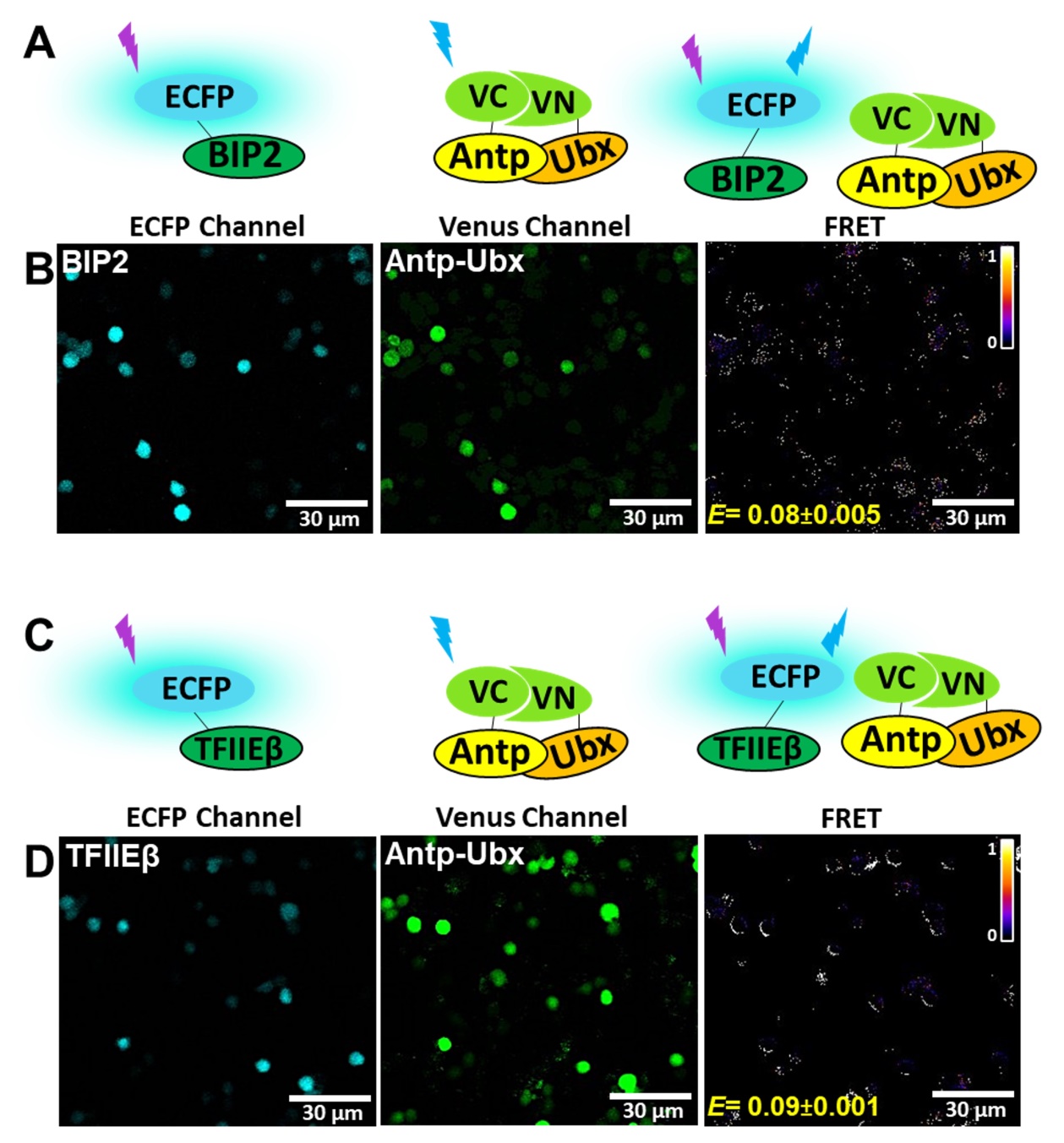


**Figure S3. BIP2 and TFIIEb do not form a trimeric interaction with Antp-Ubx by BIFC-FRET.** A) Schematic representation of BIP2 fused to ECFP (ECFP Channel), BiFC by Antp-Ubx interaction (Venus channel) and absence of energy transfer with BIP2 and Antp-Ubx heterodimer (FRET panel). B) BIP2 does not interact in a trimeric way with Antp-Ubx heterodimer (*E* = 0.08 ± 0.005). C) Schematic representation of TFIIEβ fused to ECFP (ECFP Channel), BiFC by Antp-Ubx interaction (Venus channel), and absence of energy transfer with TFIIEβ and Antp-Ubx heterodimer (FRET panel). D) TFIIEβ does not form a trimeric complex with the Antp-Ubx heterodimer (*E* = 0.09 ± 0.001). Color bar represents FRET intensity (Fire mode); lighter colors indicate high trimeric interaction levels meanwhile darker colors indicate low trimeric interaction level. Scale bar, 30 μm.
